# Exploring structural connectivity alterations in CRPS: Insights from diffusion imaging and clinical correlates

**DOI:** 10.1016/j.ynpai.2026.100234

**Published:** 2026-10-03

**Authors:** Martin Domin, Sebastian Strauss, Martin Lotze

**Affiliations:** a Functional Imaging Unit, Institute of Diagnostic Radiology and Neuroradiology, University Medicine Greifswald, Greifswald, Germany; b Department of Neurology, University Medicine Greifswald, Greifswald, Germany

**Keywords:** DTI, TBSS, Diffusion weighted imaging, CRPS, Chronic pain, Structural connectivity

## Abstract

In recent years, functional and structural brain imaging studies have accumulated heterogeneous evidence for brain-wide changes accompanying complex regional pain syndrome (CRPS). We tested possible white matter pathologies in patients with CRPS in the chronic stage in comparison to matched healthy controls. We investigated possible alterations of structural connectivity using multi-directional diffusion-weighted imaging (MDDW) in a sample of 25 patients with unilateral upper limb CRPS and 19 matched controls. In a global approach we performed tract-based spatial statistics (TBSS) to evaluate possible structural differences between groups and/or association with clinical parameters (disease duration, pain intensity, CRPS severity score/CSS, somatosensory and motor function) for the patient group. In a hypothesis-driven approach (hypotheses were predefined in a trial), we tested differences between groups for fractional anisotropy (FA) in specific tracts (thalamus-M1/S1; accumbens-thalamus; anterior insula- PFC; corpus callosum between primary hand motor representations) and associations of FA and clinical parameters. We found no significant differences between patients and HCs for both approaches. However, when calculating associations between corpus callosal tract quantification and clinical parameters we detected decreased fractional anisotropy in association with disease persistence: the longer CRPS symptoms persisted, the lower callosal white matter quantification values between both M1 upper limb representations. Further studies have to address altered interhemispheric interaction with clinical features and the possible impact for new treatment strategies.

## Highlights

• We tested possible white matter pathologies between CRPS-patients and HCs
• We used whole brain and region of interest based white matter quantification.
• There were no differences between CRPS-patients and HCs for both methods.
• Callosal fibers between M1 were associated in FD with duration of disease.
• Analyses were predefined in a trial (https://osf.io/e37td/).

## 1 Introduction

Complex regional pain syndrome (CRPS) develops in around 8% of patients with severe injuries to the upper extremity (Beerthuizen et al., 2012). The syndrome is characterized by chronic pain, allodynia (Kupers et al., 2006), autonomic dysfunction (Veldman et al., 1993), impairments in perception (Galer et al., 2013; Pleger et al., 2006; Reinersmann et al., 2021), and motor dysfunction (Azqueta-Gavaldon et al., 2017; Birklein and Rowbotham, 2005; Maihofner et al., 2007). CRPS is characterized by specific changes in the CNS which are related to disease duration and intensity of symptoms. These comprise decrease in intracortical inhibition of the hemisphere contralateral to the affected hand in the primary motor cortex/M1 (Strauss et al., 2015) and the primary somatosensory cortex/S1 (Lenz et al., 2011), changes in somatotopic representational order in the affected hand area (Pfannmöller et al., 2019), and changes in fMRI-activation in S1 and anterior insula (Geha et al., 2008). Also structural pathologies have been described: a decrease in gray matter was reported in several cortical and subcortical brain structures including the medial thalamus (Domin et al., 2021), the insula (Geha et al., 2008; Domin et al., 2021; Barad et al., 2014), nucleus accumbens (NAc) (Geha et al., 2008), the anterior cingulate cortex (ACC), the medial prefrontal cortex (PFC) (Geha et al., 2008; Domin et al., 2021; Barad et al., 2014), M1 [20] and the putamen (Azqueta-Gavaldon et al., 2017).

Especially reports about functional and structural connectivity abnormalities are highly diverse which might be related to differences in clinical samples, differences in measurement and data evaluation methods but also to a lack of sufficient statistical power of sample sizes.

With respect to structural connectivity, Geha and colleagues (Geha et al., 2008) reported decrease in fractional anisotropy (FA) in the left cingulum-callosal bundle interconnecting the ACC and supplementary motor area (SMA). They also observed increased structural connectivity between the ventromedial PFC (VMPFC) and the right anterior insular cortex (AIC), as well as decreased connectivity between VMPFC and basal ganglia. Hotta et al. (Hotta et al., 2017) reported wide-spread alterations in white matter integrity, including decreased overall FA and increased mean diffusivity in CRPS patients especially for the corpus callosum. More recently, Im and colleagues (Im et al., 2021) observed decreased prefrontal FA in CRPS patients as well as a negative association between FA and pain catastrophizing. Overall, FA-quantification of white matter tracts have been superior to other tract-based quantification methods in prior methodological studies comparing associations of FA-quantification methods with clinical characteristics (Domin et al., 2014).

Given these different methodological approaches we used an exploratory whole brain approach and a hypothesis driven approach for testing group differences of white matter structure between CRPS patients and healthy matched controls (HCs). In addition, we expected associations between symptom severity (pain intensity), sensorimotor impairment (Roeder test, spatial tactile resolution) and duration of the disease and quantification of connectivity in the preselected tracts. These procedures had been predefined and published in an on-line trial description.

## 2 Method

### 2.1 Participants and recruitment

In total, 28 patients with CRPS and 19 healthy controls (HCs) were included at the University Medicine of Greifswald, Germany. Inclusion criteria for the patient group was unilateral CRPS (for diagnostic criteria see (Harden et al., 2007)) of the upper limb for more than 3 months and absence of other neurological and psychiatric diseases (e.g., major depression). Three patients were excluded due to withdrawal. The demographic characteristics of patients and controls are plotted in Table 1. The study protocol was approved by the Ethics committee of the University Medicine of Greifswald, Germany (BB 055/18) and was conducted in accordance with the tenets of the Declaration of Helsinki. All participants provided their written consent before participating in this study. Analysis procedures had been predefined in a trial (https://osf.io/e37td/) before starting analyses (besides of the CC-quantification and corresponding regression analysis). The sample had been analyzed for other imaging parameters before (Hok et al., 2024).

**Table 1:** Participants´ characteristics.

	CRPS patients	Healthy controls	*t*-tests
Number	25	19	
Age in years	51.5 ± 14.9	51.5 ± 16.1	t (Bach et al., 2014)=0.001; n.s.
Affected side [Left/Right]	6/19	none	
CSS^1^	11.7 ± 3.2	–	
CRPS duration [months]	59.2 ± 33.8	–	
Pain rest	5.1 ± 2.5	none	
Pain movement	6.6 ± 2.6	none	
Roeder Manipulative Aptitude Test^2^ [affected side]	102.2 ± 62.3	43.9 ± 9.5	t (Smith et al., 2006)=4.50; *p* < 0.001
TPD^3^ [mm; affected side]	3.4 ± 1.9	2.2 ± 0.7	t (Avanzino et al., 2014)=2.24; *p* = 0.031

1 CSS: CRPS severity score

2 Lafayette Instrument Company, Lafayette, IN, USA

3 TPD: Two-point discrimination

### 2.2 Testing

Actual resting pain level was assessed immediately before the magnetic resonance imaging (MRI) session using a 10-cm visual analogue rating scale, anchored at left with “not at all” and at right with “unbearable”. Pain during movement of the affected limb (5 times fist clenching in an individually tolerable force and frequency) was additionally recorded. Symptom severity was determined using the CSS (Harden et al., 2010). Two-point-discrimination (TPD) was tested using a wheel-discriminator (Sensidisk, Hannover, Germany) on the thumb of the affected hand in a decreasing order of space intervals from 15 mm–1 mm while participants were blindfolded.

### 2.3 MRI-measurement

We used a Siemens Verio 3 T equipped with a standard 32 channel head coil. To prevent motion, the head was immobilized with foam cushions. A high resolution T1-weighted structural image was acquired using Magnetization-Prepared Rapid Gradient-Echo sequence for anatomical reference (repetition time (TR) = 1690 ms, echo time (TE) = 2.52 ms, flip angle = 9°, acquisition matrix = 256 × 256, slices = 173, voxel size = 1 × 0.98 × 0.98 mm^3^). For multi-directional diffusion-weighted imaging the following sequences and parameters were used: CMRR multiband diffusion-weighted sequence (TR = 5775 ms, TE = 108 ms, flip angle = 90°, acquisition matrix = 140 × 140, slices = 90, voxel size = 1.5 × 1.5 × 1.5 mm^3^, multiband acceleration factor = 3, diffusion-weighted volumes = 64, diffusion weighting factor b = 1000s/mm^2^; for the purpose of distortion correction, opposite phase encoding (PE) images without diffusion weighting were acquired.

### 2.4 MRI data evaluation

Raw data were converted and sorted into a BIDS-compliant directory structure. FSL’s Topup (Andersson et al., 2003) was used to correct the diffusion-weighted data for susceptibility-induced distortions by combining two opposite phase encoding directions. Afterwards, FSL’s Eddy (Andersson and Sotiropoulos, 2016) was used to correct for eddy current-induced distortions and subject movements, as well as outlier detection and their replacement. The T1w structural images were skull-stripped by antsBrainExtraction (Avants et al., 2011) (ANTs v.2.3.5) to enhance registration procedures and registered to the corrected diffusion-weighted images and the MNI ICBM 152 6th gen. Template using antsRegistration. For spatial normalization into MNI template space a concatenated transformation as well as its inverse were calculated from DWI subject space into MNI template space later to be used for transformation of atlases and ROIs from MNI space into individual subject's diffusion space. FSL’s slicesdir was used in an automatized way to create pictures for a visual quality check of registration steps (T1w->b0, MNI template->T1w, b0->T1w->MNI). In order to obtain diffusion-tensor-based metrics such as fractional anisotropy FSL’s dtifit was used to calculate the diffusion tensor. For additional quality checking an automatized rendering was created depicting a color-coded picture of the 1st Eigenvector image, modulated by FA.

For tract-based spatial statistics (Smith et al., 2006) the preprocessed diffusion data was further processed by DTI-TK (Zhang et al., 2007) in order to obtain optimally spatially aligned and normalized fractional anisotropy images. For this purpose, the diffusion tensor output of FSL’s dtifit was converted into a DTI-TK-compliant format. DTI-TK replaces the first three steps of the FSL-TBSS pipeline, largely by leveraging the full tensor matrix information during registrations instead of only using scalar fractional anisotropy values on voxel level. After processing, spatially normalized FA images in MNI template space were available for TBSS skeletonization and voxel-wise statistical inference using FSL’s randomize (for TBSS optimized TFCE, FWE-corrected *p* < 0.05, 50,000 permutations per test). TBSS was tested because there were reports on group differences between CRPS-patients and HCs using this method before (Hotta et al., 2017).

For probabilistic tractography FSL’s bedpost (Behrens et al., 2007) was used to build up a distribution of diffusion parameters at each voxel, FSL’s probtrackx for actual tractography. The resulting tractograms were spatially filtered using an atlas that delineates the number of diffusion directions for each voxel in the brain. This approach segments the brain into three compartments, each containing the information for one, two or three trackable directions, respectively. As the diffusion tensor approach and its derivates such as the fractional anisotropy cannot ensure for valid directional information in a voxel containing more than one direction, a filtering for one-direction-only voxels prior to FA extraction reduces the variance of anisotropy measures significantly (Volz et al., 2018). The filtered tractograms were then assessed in regards to their average weighted fractional anisotropy.

### 2.5 Regions-of-interest for tractography and data extraction

As preregistered in the trial the following structural connections were calculated using FSL’s probtrackx and the respective ROI pairs as tractography seeds: thalamus (Thal; Harvard-Oxford subcortical atlas)–nucleus accumbens (NAc; Harvard-Oxford subcortical atlas; the thalamus-NAc tract was selected because of its role for pain chronification (Mansour et al., 2013)), thalamus–sensorimotor cortex (SMC; Brainnetome atlas; the thalamus-SMC was selected because of (Pleger et al., 2014)) and anterior insular cortex (AIC; Neuromorphometrics atlas)–ventromedial prefrontal cortex (VMPFC; selected because of (Geha et al., 2008)). An additional pre-calculated tract (Domin and Lotze, 2019) was used for the interhemispheric structural connectivity via the corpus callosum. Here, the M1–M1 upper limbs tract was selected, thresholded for the highest connectivity probability and masked for the corpus callosum (see Fig. 1A).

**Fig. 1:**
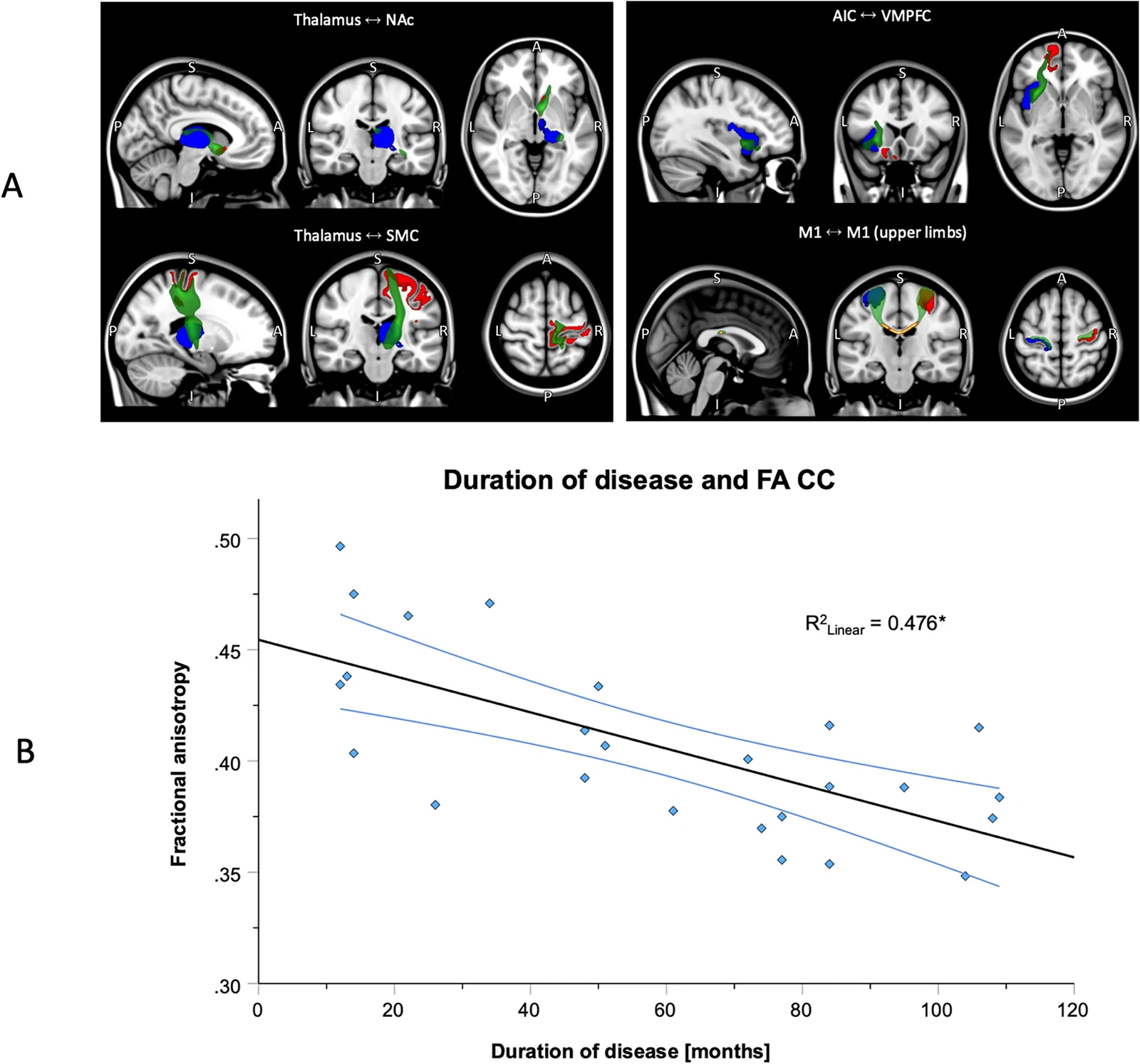
A: Fiber tracts analyzed in the diffusion-weighted imaging (DWI) pipeline: tract between thalamus and nucleus accumbens (NAC); tract between thalamus and sensorimotor cortex (SMC); tract between anterior insular cortex (AIC) and ventromedial prefrontal cortex (VMPFC); callosal tract between the M1 upper limbs regions of both hemispheres (red-yellow sub-tract depicts the assessed region). (For interpretation of the references to color in this figure legend, the reader is referred to the web version of this article.) B: Correlation plot with trendline of fractional anisotropy quantification of callosal tract (FA CC, y-axis) between both M1-upper limb areas (Domin and Lotze, 2019) and duration (time of persistence; x-axis) of CRPS in months.

### 2.6 Statistics

Potential differences in demographic and clinical variables between patients and HCs were tested using Mann-Whitney *U* test, while normally distributed variables were evaluated using two-sample *t*-test. All analyses were performed in SPSS Statistics22 (IBM, Armonk, NY, USA) with uncorrected significance level of *p* < 0.05. For FA-quantification group differences were evaluated using two-sample *t*-tests and Bonferroni-corrected for multiple comparisons by the number of fiber tracts (*n* = 4). Correlation between dependent (FA) and clinical variables (CSS, disease duration, sensorimotor function, pain intensity) was evaluated using Pearson correlation coefficient. Correlation analysis was corrected for multiple comparisons for 4 tracts and the 4 clinical values (*n* = 16). Cohen's d as an effect size measure was stated for every statistical test.

## 3 Results

### 3.1 Clinical data

Clinical parameters and group comparisons are provided in Table 1. Age was comparable between groups (t (Bach et al., 2014)=0.001; n.s). CRPS patients and healthy controls differed in TPD for the affected thumb (t (Avanzino et al., 2014)=2.24; *p* = 0.031; Cohen’s d = 0.68), and the Roeder Test for the affected hand (t (Smith et al., 2006)=4.50; *p* < 0.001; Cohen’s d = 1.37).

### 3.2 Group comparisons for white matter quantification

Global DWI-analysis: For TBSS analyses, neither a group comparison between CRPS patients and healthy controls nor associations of FA with clinical variables showed any statistically significant results. Hypothesis-driven DWI analysis: Group comparisons for tract specific comparisons showed no relevant differences (for detailed statistics see Supplementary Table 1).

### 3.3 Correlation of FA with clinical parameters

There was only one association which survived multiple comparison correction: FA_CC between both M1-upper limb areas was negatively associated with duration of CRPS (r (Smith et al., 2006)= − 0.69; non-corrected *p* < 0.001; Cohen’s d = 1.907; *p*-value corrected for 4 tracts and 4 clinical parameters (*n* = 16): p_c_ = 0.016; see Fig. 1B). The longer CRPS persisted the less FA in the corpus callosal tract between both upper limb M1 areas. This was independent of patient age (partial correlation with age as confounder: r (Andersson et al., 2003)= − 0.674; *p* < 0.001; Cohen’s d = −1.870; p_c_ = 0.016).

## 4 Discussion

This study investigated structural connectivity in patients with CRPS affecting the upper limb, employing tract-based spatial statistics and fractional anisotropy (FA) analyses to explore potential biomarkers and to address the existing heterogeneous findings in the literature. While no significant group-level differences were observed in the global or hypothesis-driven analyses, a notable negative correlation between corpus callosal FA and disease duration suggests a role for interhemispheric connectivity in the progression of CRPS. These findings provide important implications for understanding CRPS pathophysiology and highlight areas for future research.

The absence of significant group differences in TBSS metrics could reflect several factors, including the variability in CRPS phenotype and degree of motor and somatosensory symptom expression. This negative finding is in accordance to a recent other study on a comparable clinical sample on CRPS-patients in a comparable sample size (Wang et al., 2026). Our results contradict finding in earlier studies that reported reduced FA in specific tracts in CRPS. Geha et al. (Geha et al., 2008) reported decreased FA in the cingulum bundle, while Hotta et al. (Hotta et al., 2017) described widespread alterations in white matter integrity, particularly in the corpus callosum. However, both studies acknowledged methodological challenges, including variability in imaging protocols and statistical analyses although patient collectives seemed to be partially comparable. Hotta et al. (Hotta et al., 2017) investigated only 12 patients but also in the chronic stage of disease. They found associations of TBSS quantification in the callosum with motor impairments calculated by a sum of movement related pain intensity and upper-limb disability DASH (Hudak et al., 1996). We utilized the Roeder test as a direct measure to assess motor performance, specifically focusing on pinch grip strength, which is closely linked to the spatial-tactile resolution of the fingertips (Tremblay et al., 2003). In our patient cohort, we observed a relevant association between Roeder test performance and two-point discrimination. However, no significant associations were found between performance metrics and DTI parameters.

The observed association between reduced fractional anisotropy (FA) in corpus callosal pathways and prolonged disease duration suggests that interhemispheric connectivity disruptions may be a hallmark of late-stage chronic CRPS. These findings align with existing literature on other pain conditions, which also report a correlation between disease duration and reduced structural integrity of corpus callosal pathways as measured by diffusion tensor imaging (Yu et al., 2013). With respect to other symptoms related to chronic pain such as depression, anxiety and increased stress level most associations with altered connectivity point to prefrontal changes. For instance, Im et al. (Im et al., 2021) reported decreased prefrontal FA in CRPS patients as well as a negative association between FA and pain catastrophizing. Since we did not control for anxiety and stress in this patient group (but see (Klepzig et al., 2024) for craniofacial pain) we cannot add data on that associations.

The interaction between upper limb M1-representation areas might well be modified by unilateral immobilization. Even in healthy volunteers, immobilization of the upper limb by a splint for 10 h relevantly reduced interhemispheric inhibition between both upper limb M1-areas which could be avoided when using tactile stimulation (vibration) on the immobilized hand (Avanzino et al., 2014). Reduced intracortical inhibition in the upper limb area of M1 contralateral to the affected hand in CRPS patients (Strauss et al., 2015; Strauss et al., 2021) may be linked to diminished interhemispheric interactions. This imbalance likely contributes to asymmetric neurophysiological activity across the M1 representation sites of the upper limbs, as observed in prior studies, including investigations into the effects of ketamine on cortical function (Sorel et al., 2018).

Two interventional studies have demonstrated a strong association between various functional biomarkers and performance changes (Strauss et al., 2021; Gustin et al., 2010).

In contrast, structural changes seem to be less suited for using as biomarkers because if they persist, they are usually long-term changes and do not respond to short term interventions. This holds true not only for the white matter but also for the gray matter (Domin et al., 2021) suggesting that most of the pathology observed in neuroimaging studies which had been associated with performance deficits are on the functional level.

However, the potential role of corpus callosal connectivity as a biomarker for CRPS progression has clinical implications. Reduced interhemispheric connectivity could serve as an indicator of disease chronification, guiding interventions aimed at restoring neural balance. Non-invasive neuromodulation techniques could be explored to target these connectivity disruptions and improve functional outcomes. Furthermore, understanding the relationship between structural changes and clinical parameters, such as pain intensity and sensorimotor impairments, may help stratify patients for personalized treatment approaches.

To advance the understanding of complex regional pain syndrome (CRPS), future research should prioritize longitudinal study designs to monitor changes in structural connectivity over time. Such designs are essential for disentangling pathophysiological processes from behavioral adaptations and pre-existing vulnerabilities. Integrating multimodal methodologies—such as diffusion imaging, functional MRI, and electrophysiological techniques—will further enhance the capacity to elucidate the interplay between structural and functional connectivity in CRPS.

Given the considerable heterogeneity of CRPS in terms of symptom severity, disease duration, and comorbid conditions, stratification of study populations based on clinical phenotypes is essential to uncover subgroup-specific mechanisms and therapeutic responses. In this context, future large-scale, multicenter studies should follow the recent recommendations of the OptiMeth-CRPS Network Group, which emphasize the importance of patient subtyping along dimensions such as psychosocial factors, symptom characteristics, and distinct pathophysiological profiles (Smart et al., 2025). Such an approach holds promise for identifying clinically relevant effect modifiers and ultimately supports the development of more personalized, mechanism-based treatment strategies.

### 4.1 Limitations

We here selected two different quantification methods for white matter connectivity strength. The TBSS- analysis is a rather explorative approach whereas the FA-analyses were based on prior findings on these patients. Although fractional anisotropy is a robust and widely used diffusion metric it suffers from the inability of the diffusion tensor model of resolving crossing fibers which influence the FA estimation. We here tried to mitigate this issue by filtering the voxels with an atlas mask describing voxels containing only one primary diffusion direction. This highly depends on successful spatial registration processes between the individual subject and the MNI template space. Furthermore, TBSS does show limited validity especially when comparing tracts between different samples (Bach et al., 2014). There are several factors which might have an impact on white matter quantification which was not inserted in our analysis. For sex, with respect of group differences both groups, these showed comparable sex distribution (CRPS: 76% female; HCs: 68% female). For the body mass index (BMI) that was not evaluated in our sample although an impact on white matter quantification had been reported before (Repple et al., 2018). In addition, head movements were not accounted for statistical models but might have an impact on white matter quantification, too.

It has to be mentioned that other factors such as medication might have an impact on the positive findings of the correlation of FA in callosal M1 fibers with disease duration. On the other hand, negative findings might be influenced by the heterogenic sample investigated i.e., with respect to disease duration. Medication was recorded but sample size limits further subdivisions of groups. Therefore, the impact of medication might be tested in larger samples.

Although we predefined our procedures in a trial, we did not include the calculation of associations between FA obtained in the upper limb M1-M1 callosal fibers. Since any other effects were not reaching significance, this finding has to be reproduced in another sample.

In addition, sample size might have an impact on negative findings in group comparisons. However, other groups did also not find relevant differences for white matter quantification between patients with CRPS and healthy matched controls (Wang et al., 2026).

## 5 Conclusion

This study provides evidence for an association between structural connectivity alterations in the corpus callosum and CRPS duration, suggesting a potential role for interhemispheric disinhibition in disease chronification. While group-level differences in structural connectivity were not observed, these findings underscore the importance of interhemispheric pathways in CRPS pathophysiology. Future research should focus on longitudinal and multimodal approaches to validate these findings and explore their implications for targeted interventions. For outcome prediction and decision for most promising interventional strategies, multimodel fusion models with integration of structural integrity, as it had been performed for upper limb recovery following stroke (Stinear et al., 2012), might however be promising.

## CRediT authorship contribution statement

**Martin Domin:** Writing – review & editing, Software, Investigation, Data curation. **Sebastian Strauss:** Writing – review & editing, Writing – original draft, Validation, Resources, Conceptualization. **Martin Lotze:** Writing – review & editing, Writing – original draft, Supervision, Resources, Funding acquisition, Conceptualization.

## Declaration of competing interest

The authors declare that they have no known competing financial interests or personal relationships that could have appeared to influence the work reported in this paper.

## Data Availability

Data will be made available on request.
