# Supplementary material for: Exploring structural connectivity alterations in CRPS: Insights from diffusion imaging and clinical correlates
Source: Neurobiol Pain. 2026 Oct 3;20:100234. doi: 10.1016/j.ynpai.2026.100234 (PMC13677330; doi:10.1016/j.ynpai.2026.100234)
Supplement: Supplementary file 1 [file mmc1.docx]

Supplementory Table 1: FA-differences between groups

|  | **CRPS**  **patients** | **Healthy**  **controls** | **t-tests** | **Effect size** |
| --- | --- | --- | --- | --- |
| **Callosal M1 – M1 connection** | 0.41 ± 0.04 | 0.40 ± 0.04 | t(42) = -0.59; n.s. | Cohen’s d = -0.18 |
| **Anterior insula – PFC [affected side]** | 0.42 ± 0.02 | 0.42 ± 0.04 | t(42) = 0.27; n.s. | Cohen’s d = 0.08 |
| **Thalamus – NAc [affected side]** | 0.52 ± 0.02 | 0.51 ± 0.02 | t(42) = 1.81; n.s. | Cohen’s d = 0.55 |
| **Thalamus – M1/S1 [affected side]** | 0.43 ± 0.03 | 0.43 ± 0.04 | t(42) = -0.02; n.s. | Cohen’s d = -0.01 |
